# Investigating Taste Perception of Maltodextrins Using Lactisole and Acarbose

**DOI:** 10.3390/foods13132130

**Published:** 2024-07-04

**Authors:** Claudia Hartley, Russell S. J. Keast, Amelia J. Carr, Spencer S. H. Roberts, Wender L. P. Bredie

**Affiliations:** 1 CASS Food Research Centre, Deakin University, Burwood Highway, Burwood, VIC 3125, Australia; c.hartley@deakin.edu.au (C.H.); russell.keast@deakin.edu.au (R.S.J.K.); 2 Department of Food Science, University of Copenhagen, Rolighedsvej 26, 1958 Frederiksberg, Denmark; 3 Centre for Sport Research, Institute for Physical Activity and Nutrition, Deakin University, Geelong, VIC 3220, Australia; amelia.carr@deakin.edu.au (A.J.C.); s.roberts@deakin.edu.au (S.S.H.R.)

**Keywords:** complex carbohydrates, taste perception, degree of polymerisation, lactisole, acarbose, maltodextrins

## Abstract

Previous research has demonstrated that complex carbohydrates (maltodextrins) can be perceived in the oral cavity. However, little research has been conducted to thoroughly investigate complex carbohydrate taste perception and contributing factors. This study explored the effects of the degree of polymerization and the concentration of complex carbohydrates on taste perception. Additionally, the impact of lactisole and acarbose on carbohydrate taste perception was investigated. Using a blinded, Latin Square design, participants (*n* = 40) received samples (control) or samples with acarbose (5 mM) or lactisole (1.4 mM). Per visit, participants received solutions: (1) short chain maltodextrin (average DP 6) (SCM), (2) long chain maltodextrin (average DP 24) (LCM), (3) maltose, and (4) glucose. Samples were evaluated in duplicate, both at low concentration and high concentration. Participants tasted the samples and rated sweetness, starchiness, and viscosity (mouthfeel) perceived on a 10 cm continuous line scale and perceived intensity on a Labelled Magnitude Scale. There was a significant effect of degree of polymerisation on sweetness (*p* = 0.001) and intensity (*p* = 0.001). For low concentration samples, no significant differences were found between LCM and acarbose LCM or SCM and acarbose SCM for sweetness, starchiness, or mouthfeel (all *p* > 0.05). Significant differences were observed between LCM and lactisole LCM for sweetness (1.1 ± 0.1 vs. 2.5 ± 0.3, *p* = 0.001), starchiness (1.4 ± 0.2 vs. 2.3 ± 0.3, *p* = 0.005), and mouthfeel (1.4 ± 0.2 vs. 2.3 ± 0.3, *p* = 0.013). In conclusion, the taste perception of maltodextrins is influenced by the degree of polymerisation. Furthermore, for this study, the sweet taste receptor was not involved in maltodextrin taste perception. While salivary α-amylase did not appear to influence taste perception with low concentration maltodextrins, further investigation is necessary.

## 1. Introduction

Carbohydrates are staples in the human diet as they represent a major source of energy [1,2]. Current dietary recommendations suggest that adults should obtain 45–65% of their total calorie intake from carbohydrates [3]. Carbohydrates can be divided into categories based on chemical structure: (1) simple sugars (mono/disaccharides) and (2) complex carbohydrates (oligosaccharides and polysaccharides) [2,4]. Oligosaccharides are complex carbohydrates with a degree of polymerisation (DP) of 3–20, while polysaccharides are complex carbohydrates with a DP of 21 or greater. Digestible oligosaccharides and polysaccharides include starch, which is a primary source of energy and is required to maintain proper bodily function [5]. Maltodextrin, a commercially available starch hydrolysis product, is commonly used as a stabilizing or thickening agent in food and beverage products [6]. Maltodextrins are generally considered tasteless [7] and non-sweet [8] and are often composed of a combination of maltooligosaccharides and maltopolysaccharides [4].

Taste perception of simple sugars is known to occur at the sweet receptor, T1R2/T1R3 [9,10], and it was thought that complex carbohydrates were invisible to the human palate [11,12]. However, research has determined that complex carbohydrates can be perceived in the oral cavity, independent of sweet taste [13,14,15]. Furthermore, Low, et al. [16] established that participants who were more sensitive to carbohydrate taste had an increased waist circumference and a greater energy and starch intake, in comparison with participants who were less sensitive to carbohydrate taste. Additionally, studies have shown that complex carbohydrate samples can elicit a distinct “starchy” taste, characterised by descriptors such as “bread-”, “cereal-”, or “rice-like”, which can be differentiated from the sweet taste of sugars [17]. This confirms that complex carbohydrates can be sensed in the oral cavity and that they can produce the taste quality of starchiness.

Dextrose equivalent (DE) and degree of polymerisation (DP) are a structural factor that can influence the taste properties of the maltodextrins. Dextrose equivalent refers to the amount of reducing sugars present relative to the total carbohydrate count [18], while the degree of polymerisation refers to the number of linked units in a saccharide [18]. For example, a shorter chain maltodextrin has a higher DE and lower DP. Therefore, it has a sweeter taste in comparison to a longer chain maltodextrin [19]. In contrast, longer chain maltodextrins with a lower DE and higher DP elicit a more savoury taste [20] and viscous mouthfeel [8,21]. Additionally, two types of glucose polymers make up starches: amylopectin and amylose [4]. The ratio between amylopectin and amylose can affect the physical properties of starches, including their viscosity, retrogradation tendencies, and pasting properties [22,23,24]. At present, little research has thoroughly assessed differences in taste perception between maltodextrins of varying DPs and DEs. Research by Martin, et al. [25] did investigate the taste detection of various maltooligosaccharides at different concentrations with 5 mM acarbose. Using difference testing and a tongue-swabbing technique, they found that humans are able to detect maltooligosaccharides of all chain lengths. However, to our knowledge, no existing research has investigated how the perceived sweetness, starchiness, mouthfeel, and intensity differ for maltodextrins of varying degrees of polymerisation.

In taste research, various inhibitors, including lactisole and acarbose, are often used while investigating carbohydrate discrimination and taste perception. Lactisole is a sweet taste inhibitor that binds to the transmembrane region of the T1R3 [26]. It has been used in previous research by Pullicin, et al. [27] and Lapis, Penner, and Lim [14] to investigate carbohydrate taste perception and to investigate if the sweet taste receptor (T1R2/T1R3) is involved in carbohydrate taste perception. Although tasteless itself, lactisole has been known to elicit a sweet “water taste” when rinsed away from the receptor, [28] making it necessary to serve lactisole-containing solutions at colder temperatures to minimise this effect [29]. Acarbose is an effective salivary α-amylase inhibitor [30]. Acarbose has been used in previous taste research to maintain the saccharide profile of the carbohydrate sample [14,27]. Acarbose consists of an acarviosin moiety α-1,4-linked to a maltose, which simulates the transition state for the enzymatic cleavage of glycosidic linkages, enabling it to effectively inhibit salivary α-amylase [4,31]. Salivary α-amylase, the predominant enzyme in human saliva [32], plays a crucial role in the rapid modification of starch’s physical properties [33]. Once food enters the oral cavity, salivary α-amylase initiates the hydrolysis of α-1,4 glycosidic linkages in carbohydrates and starches, resulting in the production of maltose (DP 2), maltotriose (DP 3), and larger oligosaccharides [34].

Despite this previously conducted research, several gaps remain in the research area. Specifically, there is a lack of comprehensive investigations into the differences in taste perception among maltodextrins with varying DPs and DEs. Additionally, little is known about how altering the concentration of maltodextrin samples can affect taste perception (perceived sweetness, starchiness, mouthfeel, and intensity). Therefore, this paper will investigate the effect of DP and DE and concentration on carbohydrate taste perception. Furthermore, this paper aims to further investigate how the use of the sweet taste inhibitor, lactisole, and the salivary α-amylase inhibitor, acarbose, can affect carbohydrate taste perception.

## 2. Materials and Methods

### 2.1. Participants

Forty participants (10 males, 30 females; BMI [kg/m^2^]: 24.08 ± 0.99, age [years]: 30.40 ± 1.24) completed the study. Participants were recruited from the University of Copenhagen and surrounding areas across Copenhagen, Denmark. Prior to participation, each participant provided their written, informed consent, and the protocol was approved by the Deakin University Human Research Ethics Committee (project number: 2022-281) and the University of Copenhagen Ethics Committee (project number: 514-0356/22-5000). This study was conducted in accordance with the Declaration of Helsinki.

For this study, the inclusion criteria comprised of: participants aged 18–50. Participants were excluded from participation in this study if they: (1) were smokers; (2) had known food allergies; (3) had known impaired smell or taste function; and (4) were pregnant or lactating. Participants were instructed to fast for minimum of 1 h prior to all sessions.

### 2.2. Experimental Design

Participants attended a total of three sessions in the sensory laboratory at the University of Copenhagen, Frederiksberg campus. Participants arrived in a fasted state, ready for the session to begin. Between each session, participants had a minimum 48 h washout period. The sessions were conducted in a blinded, cross-over, randomised, Latin Square design. As per the Latin Square design, per session, participants were randomly allocated the conditions of: (1) samples (control); (2) samples with 1.4 mM lactisole; or (3) samples with 5 mM acarbose.

### 2.3. Sensory Stimuli

Four carbohydrate stimuli (two maltodextrins (short chain maltodextrin (SCM): average DP 6, C*Dry MD 01915, Cargill, Haubourdin, France; long chain maltodextrin (LCM): average DP 20, C*Dry MD 01955, Cargill, Haubourdin, France), glucose (Melbourne Taste Depot, Melbourne, VIC, Australia), and maltose (Thermo Scientific Chemicals, Minato-Ku, Tokyo, Japan) were used to investigate carbohydrate taste function. Samples were presented at a low concentration and at a high concentration in duplicate. For further details of stimuli, see Table 1. To further investigate the taste perception of carbohydrates, a sweet taste inhibitor, lactisole (Cayman Chemical, Ann Arbor, MI, USA), and a salivary α-alpha amylase inhibitor, acarbose (Cayman Chemical, Ann Arbor, MI, USA), were used. Using a Latin Square design, participants received either the carbohydrate samples (control), the samples with 1.4 mM lactisole, or the samples with 5 mM acarbose [14] per visit. Acarbose was added to the maltodextrin samples (LCM and SCM) only as the acarbose would have no effect on the maltose or glucose samples. Prior to testing, solutions were prepared in glassware with filtered water and refrigerated. All solutions were served at 10 °C to minimise any sweet “water taste” from the lactisole [27].

### 2.4. Sensory Methods

All taste assessment measures were conducted in individual, computerised, partitioned sensory booths in a laboratory environment. Sensory-evaluation software Compusense Cloud version 23.0 (Compusense Inc., Guelph, ON, Canada) was used to collect the taste-assessment measures for this study. Data were generated through accessing research infrastructure at Copenhagen University, including FOODHAY (Food and Health Open Innovation Laboratory, Danish Roadmap for Research Infrastructure). All solutions were served at a temperature of 10 °C and presented in a sample cup printed with a three-digit code for blinding purposes. During the taste evaluations, participants were provided with a tray containing four 5 mL samples. They were instructed to wear a nose clip, take the entire sample into their mouth, swish it around for 5 s, and then spit it out. For each tastant, participants were asked to rate the intensity of each sample using the Labelled Magnitude Scale (LMS) [35]. This scale is a vertical line scale with descriptors ranging from “barely detectable”, “weak”, “moderate”, “strong”, “very strong”, and “strongest imaginable”. Participants were also instructed to provide ratings for perceived sweetness, mouthfeel, and starchiness on a 10 cm continuous line scale (scale ranges from 0 = no sensation to 10 = extremely strong sensation). Between samples, participants rinsed their mouths with the filtered water provided as an oral rinsing agent [15]. When provided lactisole-containing samples, participants were instructed to not rinse their mouth between samples to avoid any sweet “water taste” [14,29]. For an oral rinsing agent, filtered water was used [15].

### 2.5. Statistical Analysis

Statistical analysis was performed using Stata Statistical software version 16.0 (StataCorp LLC, College Station, TX, USA). Data are presented as mean with standard errors of mean (SEM). Statistical significance was accepted at *p* < 0.05 for all analyses. Participants used a 10 cm continuous line scale to rate perceived sweetness, mouthfeel, and starchiness. To account for end-of-scale use avoidance, the scale was extended to −1 and 11. During analysis, the data were transformed to a 12 cm continuous line scale to account for this. The effect of the treatment (lactisole or acarbose), stimuli, and concentration on the outcomes (taste intensity and ratings of sweetness, mouthfeel, and starchiness) were estimated using linear mixed models with the variables of the treatment and period (the order each treatment was received) as fixed effects and the participant as a random effect variable. To examine further differences between samples, paired *t*-tests were used.

## 3. Results

### 3.1. Effect of Tastant on Taste Perception

#### 3.1.1. Differences between LCM and SCM Samples

Overall, for the LCM and SCM samples, there was a significant effect of maltodextrin chain length and structure on sweetness (*p* = 0.001) and intensity (*p* = 0.001). There was no effect of maltodextrin chain length on mouthfeel (*p* = 0.709) or starchiness (*p* = 0.914). Comparing the LCM and SCM samples, there was a mean difference of −0.43 (*p* = 0.001) in sweetness and −2.18 (*p* = 0.001) in intensity.

At the low concentration, there were no significant differences between SCM and LCM for sweetness, starchiness, mouthfeel, or overall perceived intensity (all *p* > 0.05) (see Table 2 for an overview). At the high concentration, there was a significant difference between SCM and LCM for sweetness. Additionally, when comparing the LCM and SCM samples, the SCM sample was perceived to be more intense overall. There were no significant differences between the samples for starchiness or mouthfeel (all *p* > 0.05).

#### 3.1.2. Differences between LCM and SCM Samples, Glucose, and Maltose

Overall, for all samples, there was a significant effect of structure on sweetness (*p* = 0.001), mouthfeel (*p* = 0.001), and intensity (*p* = 0.001). There was no effect of structure on starchiness (*p* > 0.05).

At the low concentration, there were significant differences between LCM and glucose samples. In comparison to the LCM sample, the glucose sample was perceived to be sweeter, have a greater starchiness, overall increased mouthfeel, and greater perceived intensity (Table 3). Similarly, at the low concentration, in comparison to the SCM sample, the glucose sample was perceived to be sweeter, have a greater starchiness, overall mouthfeel, and perceived to be more intense overall (Table 4).

At the high concentration, there was a significant difference between LCM and glucose in sweetness, mouthfeel, and overall intensity (Table 3). There were no significant differences between the samples for starchiness (*p* > 0.05). At the high concentration, there were significant differences between SCM and glucose in sweetness, mouthfeel, and overall intensity (Table 4). There were no significant differences between the samples for starchiness (*p* > 0.05).

At the low concentration, there were significant differences between LCM and maltose for sweetness and overall intensity (Table 5). There were no significant differences in starchiness or mouthfeel (all *p* > 0.05). Similarly, at the low concentration, there were significant differences between SCM and maltose for sweetness, starchiness, and overall intensity (Table 6). There were no significant differences in mouthfeel (*p* > 0.05). 

Relative to the LCM sample at high concentration, the maltose samples reported significantly higher scores for sweetness and overall intensity (Table 5). There were no significant differences in starchiness or mouthfeel (all *p* > 0.05). Similarly, between SCM and maltose, there were significant differences for sweetness and overall intensity (Table 6). There were no significant differences between the samples for starchiness or mouthfeel (all *p* > 0.05).

### 3.2. Effect of Concentration on Complex Carbohydrate Taste Perception

Comparing SCM at low concentration with SCM at high concentration, there were significant differences in sweetness, starchiness, mouthfeel, and intensity (see Table 7 for an overview of the results). Similarly, when comparing LCM at low concentration with LCM at high concentration, there were significant differences in sweetness, mouthfeel, and intensity. However, there were no significant differences for starchiness (*p* > 0.05). There were further significant differences in intensity when comparing maltose at low concentration and high concentration (8.1 ± 1.0 vs. 22.9 ± 2.0, *p* = 0.0001) and glucose at low concentration and glucose at high concentration (21.8 ± 1.9 vs. 57.9 ± 2.7, *p* = 0.0001).

### 3.3. Effect of Lactisole on Taste Perception

#### 3.3.1. Effect of Lactisole on Overall Sweetness

At low concentration, for samples of a lower sweetness (SCM, LCM, and maltose samples), the addition of lactisole significantly increased perceived sweetness (all *p* < 0.05, Table 8). Contrastingly, for the sweetest sample, glucose, the addition of lactisole significantly reduced the perceived sweetness (4.4 ± 0.3 vs. 2.3 ± 0.2, *p* = 0.0001). This was similar at high concentration where the perception of sweetness was significantly reduced with lactisole for glucose and maltose samples only (*p* = 0.0001 and *p* = 0.0001, Table 8).

#### 3.3.2. Effect of Lactisole on Complex Carbohydrate Taste Perception

Overall, for the LCM and SCM samples, there was a significant effect of the treatment (lactisole) on sweetness (*p* = 0.001), mouthfeel (*p* = 0.001), starchiness (*p* = 0.001), and intensity (*p* = 0.001).

At the low concentration, there were significant differences between the control SCM sample and the lactisole SCM sample for sweetness (1.3 ± 0.1 vs. 2.6 ± 0.3, *p* = 0.001) and starchiness (1.3 ± 0.2 vs. 2.0 ± 0.2, *p* = 0.009). There were no significant differences in mouthfeel (*p* > 0.05). When comparing the control LCM sample and the lactisole LCM sample at the low concentration, there were significant differences for sweetness (1.1 ± 0.1 vs. 2.5 ± 0.3, *p* = 0.001), starchiness (1.4 ± 0.2 vs. 2.3 ± 0.3, *p* = 0.005), and mouthfeel (1.4 ± 0.2 vs. 2.3 ± 0.3, *p* = 0.013). Furthermore, at the high concentration, there were no significant differences between the control SCM sample and the lactisole SCM sample for sweetness, starchiness, and mouthfeel (all *p* > 0.05). However, when comparing the control LCM sample to the lactisole LCM sample at the high concentration, there was a significant difference for sweetness (1.3 ± 0.1 vs. 2.2 ± 0.2, *p* = 0.001). There were no significant differences in starchiness or mouthfeel (all *p* > 0.05).

### 3.4. Effect of Acarbose on Complex Carbohydrate Taste Perception

Overall, for the LCM and SCM samples, there was a significant effect of the treatment (acarbose) on sweetness (*p* = 0.001), mouthfeel (*p* = 0.001), starchiness (*p* = 0.001), and intensity (*p* = 0.001).

When comparing the control SCM sample to the acarbose SCM sample at the low concentration, there were no significant differences in sweetness, starchiness, or mouthfeel (*p* > 0.05, Table 9). Similarly, at the low concentration, comparing the control LCM sample and the acarbose SCM sample, there were no significant differences for sweetness, starchiness, and mouthfeel (all *p* > 0.05, Table 10). At the high concentration, there were significant differences between the control SCM samples and the acarbose SCM samples. There were significant differences with sweetness and starchiness (Table 9). There were no significant differences with mouthfeel (*p* > 0.05). However, when comparing the control LCM samples and the acarbose LCM samples at the high concentration, there were no significant differences in sweetness, starchiness, or mouthfeel (all *p* > 0.05, Table 10).

## 4. Discussion

This study investigated the effect of maltodextrin structure (DP and DE) and concentration on carbohydrate taste perception. Further, this study investigated how sweet taste inhibitor, lactisole, and salivary α-amylase inhibitor, acarbose, influence carbohydrate taste perception. While previous literature has investigated the discriminability and detection of complex carbohydrates [14,15,27], as well as taste intensity of complex carbohydrate stimuli [13], to the best of our knowledge, this is the first study to investigate the taste perception of complex carbohydrates alongside the factors of structure, concentration, and various inhibitors.

The results demonstrated that taste perception of maltodextrins can be influenced by chain length and degree of polymerisation. When the LCM and SCM samples were compared, there was a significant difference in sweetness and overall intensity (all *p* < 0.001). However, there were no significant differences between the samples for mouthfeel or starchiness (*p* > 0.05). This is consistent with previous literature from our research group that found that when assessing suprathreshold intensity perception, there was a statistically significant difference in perceived intensity between LCM and SCM samples (7.3 ± 1.1 vs. 13.7 ± 1.7, *p* = 0.0001, respectively) [36]. It was hypothesised that due to the SCM sample’s higher DE value and, therefore, higher glucose content, this could increase the perceived “sweet-like” taste and therefore, have a higher perceived intensity than the LCM sample [36]. Interestingly, there were no differences in perceived mouthfeel between the LCM and SCM samples. It was hypothesised that the LCM sample would be perceived as significantly more viscous and have an increased mouthfeel than the SCM sample. In previous research, Johnson and Srisuthep [8] measured the viscosity of linear oligosaccharides of DP 1–7 and demonstrated that viscosity increased approximately linearly with DP. An explanation could be that there are differences in measured viscosity between the LCM and SCM samples. However, any differences in perceived viscosity and mouthfeel are minor and not perceptible in the oral cavity. Furthermore, in comparison to the LCM and SCM samples, overall, the glucose samples were perceived to have a significantly increased sweetness, overall intensity, and mouthfeel (all *p* < 0.05). It is uncertain why in comparison to the maltodextrin samples, the glucose sample would have a significantly increased mouthfeel. The authors hypothesise that it could be due to multiple factors such as heat transfer in the oral cavity or a trigeminal or tactile effect. Although all solutions were served at 10 °C (to minimise any sweet “water taste” from the lactisole [14,29]), it is plausible that this increased the perceived mouthfeel of the glucose sample but did not affect the mouthfeel of the maltodextrin samples. Furthermore, it is possible that the interaction between the temperature receptors and tactile receptors in the oral cavity caused a feeling of increased mouthfeel for the glucose sample. Additional research is necessary in order to investigate these findings further.

The concentration of the samples was an additional factor that affected the taste perception of the carbohydrate samples. As all samples were served at a low concentration and a high concentration, this allowed for comparisons to be made between the taste perception of the samples. When comparing the low concentration and high concentration maltodextrin samples (SCM and LCM), increasing the concentration significantly increased the perceived sweetness, mouthfeel, and intensity. For the SCM sample, starchiness was also significantly increased. This follows the principle that an increase in taste molecules increases the intensity and, therefore, the overall sweetness [37,38]. Future research investigating carbohydrate taste perception should consider using multiple concentrations to further investigate any dose-response relationships.

This research adds to the existing body of literature reporting that complex carbohydrates can be perceived in the oral cavity, independently from sweet taste. When lactisole (a sweet taste inhibitor) was added to the solutions, the LCM and SCM were still perceived in the oral cavity. This is similar to previous work completed by Lapis, Penner, and Lim [14] where lactisole did not compromise the detectability of glucose oligomers and this, therefore, indicates that a mechanism other than the T1R2/T1R3 taste receptor is responsible for the perception of complex carbohydrates. Interestingly, in this study, compared to the LCM or SCM control samples, the lactisole-containing samples were mostly perceived to be significantly sweeter and starchier. In order to minimise any sweet “water taste” from the lactisole, as per previously used protocol, lactisole-containing solutions were served at 10 °C, and participants were instructed to not rinse with water between solutions [14,29]. It has been observed that when lactisole is rinsed from the receptor, it results in the sweet perception of water [28]. Therefore, the results may indicate that the lactisole compound itself may have elicited a sweet sensation. With samples with a lower sweetness, such as the maltodextrins (LCM and SCM samples), the addition of the lactisole significantly increased perceived sweetness. It is, therefore, hypothesised that the lactisole itself has a low level of sweetness. This, combined with the low level of sweetness from the samples, results in an increased overall sweetness. Whereas with a sample with a higher level of sweetness such as glucose, the low level of sweetness from the lactisole was outweighed, and this facilitates the reduction of sweetness through the inhibition of the T1R2/T1R3 receptor. Interestingly, the lactisole was only able to facilitate a reduction in sweetness, not an elimination. When examining previous studies that have investigated carbohydrates and lactisole, they investigated the discriminability of samples with and without lactisole [14]. Therefore, this is the first study to examine their corresponding taste properties. Hence, it is possible that in previous research, for samples with a low level of sweetness, the inclusion of lactisole also increased overall sweetness. Research by Deck, et al. [39] investigated the temporal and dose-response effects of lactisole on various sweeteners (ace K, aspartame, cyclamate, and NHDC). Using 0.46 mM and 0.92 mM lactisole, they found that after around 30 s, participants reported an increased sweetness with lactisole-containing samples. Deck, Behrens, Wendelin, Ley, Krammer, and Lieder [39] hypothesise that this increase in sweetness with lactisole is due to saliva secretion, and this, therefore, creates a sweet aftertaste impression. Alternatively, the authors hypothesise that the sweetness of lactisole could be due to the contamination of the lactisole compound itself during the purification process. Therefore, this cannot be excluded as a confounding factor.

For the majority of samples, the inhibition of salivary α-amylase with acarbose did not influence taste perception. At low concentration, no significant differences were observed between the LCM and SCM control samples and the acarbose-containing samples. This, therefore, demonstrates that salivary α-amylase does not affect the taste perception of low concentration complex carbohydrates. For these low concentration LCM and SCM samples, the hydrolysis byproducts that are produced by salivary α-amylase in the oral cavity are likely not at a level that is perceptible in the oral cavity. This is similar to findings from Lapis, Penner, and Lim [13]. They purport that under their experimental conditions, salivary α-amylase activity and the hydrolysis byproducts do not significantly affect the responsiveness to complex carbohydrates [13]. From this finding, it may suggest that in future taste research with low concentration maltodextrins, it is not necessary to use acarbose to preserve the saccharide profile. However, in our research, at high concentration, the acarbose-containing sample was perceived to be significantly sweeter and starchier than the control SCM sample (*p* < 0.05). As there was no effect on sweetness for the other samples, it is uncertain why significant differences were observed between the SCM sample at high concentration and the acarbose-containing sample. As it is a small effect, the authors hypothesise that this could be due to a false positive effect. Further investigation needs to be conducted to examine the effect of acarbose on salivary α-amylase with complex carbohydrate samples. Hence, our research group will be investigating this relationship in future work. For this study, there are strengths and limitations that should be considered. A strength of this study is that the methodology used a blinded, randomised, crossover, Latin Square design. Furthermore, this is the first study to investigate the impact of concentration, structure, and inhibitors on the taste perception of complex carbohydrates. Despite these strengths, limitations must also be acknowledged. This study had a sample size of *n* = 40 participants, and data were collected in duplicate. The findings from this study, therefore, provide an indication of effects. However, one should be cautious of applying these findings to the population level. The authors acknowledge that the sample was not balanced for sex. However, potential differences in taste perception between men and women were not assessed in this study.

## 5. Conclusions

In conclusion, this study demonstrated that the degree of polymerisation does influence the taste perception of complex carbohydrates. This research also demonstrated that with low concentration maltodextrins, salivary α-amylase did not appear to influence their taste perception. Further research is required to further investigate the relationship between acarbose, salivary α-amylase, and complex carbohydrates. However, these findings may impact future research with complex carbohydrates. Furthermore, this research builds upon previous research and supports that the sweet taste receptor, T1R2/T1R3, was not involved in the taste perception of the complex carbohydrates used in this study. This supports previous research that suggests that a separate taste receptor mechanism may be responsible for complex carbohydrate taste perception.

## Figures and Tables

**Table 1:** Tastant stimuli concentrations used for investigation of various carbohydrates on taste perception.

Stimuli	Concentration (%*w*/*v*)	
	Low	High
Glucose	5.3	21.2
Maltose	2.7	8.1
Maltodextrin (SCM)	3.6	11.2
Maltodextrin (LCM)	3.6	11.2

This concentration series was used in previous research [15]. The low concentration of maltose was used in previous research [27]. The high maltose concentration was established during benchtop testing. SCM—Short chain maltodextrin, LCM—long chain maltodextrin.

**Table 2:** Differences in taste perception (sweetness, starchiness, mouthfeel, and intensity) between SCM and LCM samples at low and high concentrations.

Perception	Stimuli Concentration	SCM Mean ± SEM	LCM Mean ± SEM	*p* Value
Sweetness	Low	1.3 ± 0.1	1.1 ± 0.1	0.139
Starchiness	Low	1.3 ± 0.2	1.4 ± 0.2	0.176
Mouthfeel	Low	1.4 ± 0.2	1.4 ± 0.2	0.881
Intensity	Low	5.0 ± 0.7	4.6 ± 0.7	0.619
Sweetness	High	1.8 ± 0.2	1.3 ± 0.1	0.004 *
Starchiness	High	1.6 ± 0.2	1.7 ± 0.2	0.582
Mouthfeel	High	1.8 ± 0.2	2.0 ± 0.2	0.249
Intensity	High	10.7 ± 1.3	6.2 ± 0.8	0.0004 *

Samples were rated on a 10 cm continuous line scale (scale ranges from 0 = no sensation to 10 = extremely strong sensation). Intensity was rated on a Labelled Magnitude Scale (LMS). * Indicates statistically significant difference (*p* < 0.05). SCM—Short chain maltodextrin, LCM—Long chain maltodextrin.

**Table 3:** Differences in taste perception (sweetness, starchiness, mouthfeel, and intensity) between LCM and glucose samples at low and high concentrations.

Perception	Stimuli Concentration	LCM Mean ± SEM	Glucose Mean ± SEM	*p* Value
Sweetness	Low	1.1 ± 0.1	4.4 ± 0.3	0.0001 *
Starchiness	Low	1.4 ± 0.2	1.8 ± 0.2	0.027 *
Mouthfeel	Low	1.4 ± 0.2	2.1 ± 0.3	0.0002 *
Intensity	Low	4.6 ± 0.7	21.8 ± 1.9	0.0001 *
Sweetness	High	1.3 ± 0.1	9.0 ± 0.3	0.0001 *
Starchiness	High	1.7 ± 0.2	1.9 ± 0.3	0.349
Mouthfeel	High	2.0 ± 0.2	2.6 ± 0.3	0.022 *
Intensity	High	6.2 ± 0.8	57.9 ± 2.7	0.0001 *

Samples were rated on a 10 cm continuous line scale (scale ranges from 0 = no sensation to 10 = extremely strong sensation). Intensity was rated on a Labelled Magnitude Scale (LMS). * Indicates statistically significant difference (*p* < 0.05). LCM—Long chain maltodextrin.

**Table 4:** Differences in taste perception (sweetness, starchiness, mouthfeel, and intensity) between SCM and glucose samples at low and high concentrations.

Perception	Stimuli Concentration	SCM Mean ± SEM	Glucose Mean ± SEM	*p* Value
Sweetness	Low	1.3 ± 0.1	4.4 ± 0.3	0.0001 *
Starchiness	Low	1.3 ± 0.2	1.8 ± 0.2	0.002 *
Mouthfeel	Low	1.4 ± 0.2	2.1 ± 0.3	0.0001 *
Intensity	Low	5.0 ± 0.7	21.8 ± 1.9	0.0001 *
Sweetness	High	1.8 ± 0.2	9.0 ± 0.3	0.0001 *
Starchiness	High	1.6 ± 0.2	1.9 ± 0.3	0.197
Mouthfeel	High	1.8 ± 0.3	2.6 ± 0.3	0.0004 *
Intensity	High	10.7 ± 1.3	57.9 ± 2.7	0.0001 *

Samples were rated on a 10 cm continuous line scale (scale ranges from 0 = no sensation to 10 = extremely strong sensation). Intensity was rated on a Labelled Magnitude Scale (LMS). * Indicates statistically significant difference (*p* < 0.05). SCM—Short chain maltodextrin.

**Table 5:** Differences in taste perception (sweetness, starchiness, mouthfeel, and intensity) between LCM and maltose samples at low and high concentrations.

Perception	Stimuli Concentration	LCM Mean ± SEM	Maltose Mean ± SEM	*p* Value
Sweetness	Low	1.1 ± 0.1	1.9 ± 0.2	0.0001 *
Starchiness	Low	1.4 ± 0.2	1.6 ± 0.2	0.236
Mouthfeel	Low	1.4 ± 0.2	1.5 ± 0.2	0.288
Intensity	Low	4.6 ± 0.7	8.1 ± 1.0	0.001 *
Sweetness	High	1.3 ± 0.1	4.9 ± 0.3	0.0001 *
Starchiness	High	1.7 ± 0.2	1.7 ± 0.2	0.883
Mouthfeel	High	2.0 ± 0.2	2.1 ± 0.3	0.537
Intensity	High	6.2 ± 0.8	22.9 ± 2.0	0.0001 *

Samples were rated on a 10 cm continuous line scale (scale ranges from 0 = no sensation to 10 = extremely strong sensation). Intensity was rated on a Labelled Magnitude Scale (LMS). * Indicates statistically significant difference (*p* < 0.05). LCM—Long chain maltodextrin.

**Table 6:** Differences in taste perception (sweetness, starchiness, mouthfeel, and intensity) between SCM and maltose samples at low and high concentrations.

Perception	Stimuli Concentration	SCM Mean ± SEM	Maltose Mean ± SEM	*p* Value
Sweetness	Low	1.3 ± 0.1	1.9 ± 0.2	0.0001 *
Starchiness	Low	1.3 ± 0.2	1.6 ± 0.2	0.041 *
Mouthfeel	Low	1.4 ± 0.2	1.5 ± 0.2	0.253
Intensity	Low	5.0 ± 0.7	8.1 ± 1.0	0.0005 *
Sweetness	High	1.8 ± 0.2	4.9 ± 0.3	0.0001 *
Starchiness	High	1.6 ± 0.2	1.7 ± 0.2	0.688
Mouthfeel	High	1.8 ± 0.2	2.1 ± 0.3	0.062
Intensity	High	10.7 ± 1.3	22.9 ± 2.0	0.0001 *

Samples were rated on a 10 cm continuous line scale (scale ranges from 0 = no sensation to 10 = extremely strong sensation). Intensity was rated on a Labelled Magnitude Scale (LMS). * Indicates statistically significant difference (*p* < 0.05). SCM—Short chain maltodextrin.

**Table 7:** Effect of change in concentration (low vs. high) on taste perception (sweetness, starchiness, mouthfeel, and intensity) of SCM and LCM samples.

Perception	Stimuli	Stimuli at Low Concentration Mean ± SEM	Stimuli at High Concentration Mean ± SEM	*p* Value
Sweetness	SCM	1.3 ± 0.1	1.8 ± 0.2	0.001 *
Starchiness	SCM	1.3 ± 0.2	1.6 ± 0.2	0.021 *
Mouthfeel	SCM	1.4 ± 0.2	1.8 ± 0.3	0.029 *
Intensity	SCM	5.0 ± 0.7	10.7 ± 1.3	0.0001 *
Sweetness	LCM	1.1 ± 0.1	1.3 ± 0.1	0.043 *
Starchiness	LCM	1.4 ± 0.2	1.7 ± 0.2	0.059
Mouthfeel	LCM	1.4 ± 0.2	2.0 ± 0.2	0.001 *
Intensity	LCM	4.6 ± 0.7	6.2 ± 0.8	0.024 *

Samples were rated on a 10 cm continuous line scale (scale ranges from 0 = no sensation to 10 = extremely strong sensation). Intensity was rated on a Labelled Magnitude Scale (LMS). * Indicates statistically significant difference (*p* < 0.05). SCM—Short chain maltodextrin, LCM—Long chain maltodextrin.

**Table 8:** Effect of lactisole (1.4 mM) on sweetness perception of carbohydrate samples prepared at low concentration.

Stimuli	Stimuli Concentration	Stimuli Mean ± SEM	Stimuli + Lactisole Mean ± SEM	*p* Value
SCM	Low	1.3 ± 0.1	2.6 ± 0.3	0.0001 *
LCM	Low	1.1 ± 0.1	2.5 ± 0.3	0.0001 *
Glucose	Low	4.4 ± 0.3	2.3 ± 0.2	0.0001 *
Maltose	Low	1.9 ± 0.2	2.8 ± 0.3	0.017 *
SCM	High	1.8 ± 0.2	2.2 ± 0.2	0.161
LCM	High	1.3 ± 0.1	2.2 ± 0.2	0.0007 *
Glucose	High	9.0 ± 0.3	4.8 ± 0.3	0.0001 *
Maltose	High	4.9 ± 0.3	2.0 ± 0.2	0.0001 *

Samples were rated on a 10 cm continuous line scale (scale ranges from 0 = no sensation to 10 = extremely strong sensation). * Indicates statistically significant difference (*p* < 0.05). SCM—Short chain maltodextrin, LCM—Long chain maltodextrin.

**Table 9:** Effect of acarbose (5 mM) on taste perception (sweetness, starchiness, mouthfeel, and intensity) of SCM sample at low and high concentrations.

Stimuli	Stimuli Concentration	SCM Mean ± SEM	SCM + Acarbose Mean ± SEM	*p* Value
Sweetness	Low	1.3 ± 0.1	1.2 ± 0.1	0.730
Starchiness	Low	1.3 ± 0.2	1.8 ± 0.2	0.075
Mouthfeel	Low	1.4 ± 0.2	1.5 ± 0.2	0.594
Intensity	Low	5.0 ± 0.7	6.6 ± 0.9	0.135
Sweetness	High	1.8 ± 0.2	2.5 ± 0.2	0.001 *
Starchiness	High	1.7 ± 0.2	2.4 ± 0.3	0.021 *
Mouthfeel	High	1.8 ± 0.2	1.9 ± 0.3	0.651
Intensity	High	10.7 ± 1.3	12.4 ± 1.5	0.319

Samples were rated on a 10 cm continuous line scale (scale ranges from 0 = no sensation to 10 = extremely strong sensation). Intensity was rated on a Labelled Magnitude Scale (LMS). * Indicates statistically significant difference (*p* < 0.05). SCM—Short chain maltodextrin.

**Table 10:** Effect of acarbose (5 mM) on taste perception (sweetness, starchiness, mouthfeel, and intensity) of LCM sample at low and high concentrations.

Stimuli	Stimuli Concentration	LCM Mean ± SEM	LCM + Acarbose Mean ± SEM	*p* Value
Sweetness	Low	1.1 ± 0.1	0.9 ± 0.1	0.253
Starchiness	Low	1.4 ± 0.2	1.6 ± 0.2	0.597
Mouthfeel	Low	1.4 ± 0.2	1.4 ± 0.2	0.100
Intensity	Low	4.6 ± 0.7	4.7 ± 0.7	0.958
Sweetness	High	1.3 ± 0.1	1.1 ± 0.1	0.179
Starchiness	High	1.7 ± 0.2	2.0 ± 0.2	0.343
Mouthfeel	High	2.0 ± 0.2	1.9 ± 0.3	0.787
Intensity	High	6.2 ± 0.8	6.4 ± 0.8	0.838

Samples were rated on a 10 cm continuous line scale (scale ranges from 0 = no sensation to 10 = extremely strong sensation). Intensity was rated on a Labelled Magnitude Scale (LMS). * Indicates statistically significant difference (*p* < 0.05). LCM—Long chain maltodextrin.

## Data Availability

The datasets generated during and/or analysed during the current study are available from the corresponding author upon reasonable request.

## References

[1] Vasileska A., Rechkoska G. (2012). Global and Regional Food Consumption Patterns and Trends. Procedia Soc. Behav. Sci..

[2] World Health Organization (1998). Carbohydrates in Human Nutrition: Report of a Joint FAO/WHO Expert Consultation, Rome, 14–18 April 1997.

[3] National Health and Medical Research Council (2006). Nutrient Reference Values for Australia and New Zealand Including Recommended Dietary Intakes.

[4] Lim J., Pullicin A. (2019). Oral carbohydrate sensing: Beyond sweet taste. Physiol. Behav..

[5] Holesh J.E., Aslam S., Martin A. (2024). Physiology, Carbohydrates. StatPearls.

[6] American Chemical Society Maltodextrin. https://www.acs.org/content/acs/en/molecule-of-the-week/archive/m/maltodextrin.html.

[7] Veldhuizen M.G., Babbs R.K., Patel B., Fobbs W., Kroemer N.B., Garcia E., Yeomans M.R., Small D.M. (2017). Integration of Sweet Taste and Metabolism Determines Carbohydrate Reward. Curr. Biol..

[8] Johnson J.A., Srisuthep R. (1975). Physical and Chemical Properties of Oligosaccharides. Cereal Chem..

[9] Nelson G., Hoon M.A., Chandrashekar J., Zhang Y., Ryba N.J., Zuker C.S. (2001). Mammalian sweet taste receptors. Cell.

[10] Zhao G.Q., Zhang Y., Hoon M.A., Chandrashekar J., Erlenbach I., Ryba N.J., Zuker C.S. (2003). The receptors for mammalian sweet and umami taste. Cell.

[11] Feigin M.B., Sclafani A., Sunday S.R. (1987). Species differences in polysaccharide and sugar taste preferences. Neurosci. Biobehav. Rev..

[12] Hettinger T.P., Frank M.E., Myers W.E. (1996). Are the tastes of polycose and monosodium glutamate unique?. Chem. Senses.

[13] Lapis T.J., Penner M.H., Lim J. (2014). Evidence that humans can taste glucose polymers. Chem. Senses.

[14] Lapis T.J., Penner M.H., Lim J. (2016). Humans Can Taste Glucose Oligomers Independent of the hT1R2/hT1R3 Sweet Taste Receptor. Chem. Senses.

[15] Low J.Y., Lacy K.E., McBride R.L., Keast R.S.J. (2017). Evidence supporting oral sensitivity to complex carbohydrates independent of sweet taste sensitivity in humans. PLoS ONE.

[16] Low J.Y., Lacy K.E., McBride R.L., Keast R.S.J. (2017). Carbohydrate Taste Sensitivity Is Associated with Starch Intake and Waist Circumference in Adults. J. Nutr..

[17] Towers N. (2013). The Effectiveness of Dietary Learning on Hedonic Responses to a Novel, Initially Disliked Vegetable. Master’s Thesis.

[18] Hofman D.L., van Buul V.J., Brouns F.J.P.H. (2016). Nutrition, Health, and Regulatory Aspects of Digestible Maltodextrins. Crit. Rev. Food Sci. Nutr..

[19] BeMiller J.N., BeMiller J.N. (2019). 19—Carbohydrate and Noncarbohydrate Sweeteners. Carbohydrate Chemistry for Food Scientists.

[20] Birch G.G., Azudin M.N., Grigor J.M. (1991). Solution Properties and Composition of Dextrins.

[21] Marchal L.M., Beeftink H.H., Tramper J. (1999). Towards a rational design of commercial maltodextrins. Trends Food Sci. Technol..

[22] Liu Q. (2005). Understanding Starches and Their Role in Foods. Food Carbohydrates: Chemistry, Physical Properties and Applications.

[23] Singh N., Singh J., Kaur L., Singh Sodhi N., Singh Gill B. (2003). Morphological, thermal and rheological properties of starches from different botanical sources. Food Chem..

[24] BeMiller J.N., Whistler R.L. (1996). Carboydrates.

[25] Martin L.E., Andrewson T.S., Penner M.H., Lim J. (2023). Taste Detection of Maltooligosaccharides with Varying Degrees of Polymerization. J. Agric. Food Chem..

[26] Jiang P., Cui M., Zhao B., Liu Z., Snyder L.A., Benard L.M., Osman R., Margolskee R.F., Max M. (2005). Lactisole interacts with the transmembrane domains of human T1R3 to inhibit sweet taste. J. Biol. Chem..

[27] Pullicin A.J., Penner M.H., Lim J. (2017). Human taste detection of glucose oligomers with low degree of polymerization. PLoS ONE.

[28] Galindo-Cuspinera V., Winnig M., Bufe B., Meyerhof W., Breslin P.A.S. (2006). A TAS1R receptor-based explanation of sweet ‘water-taste’. Nature.

[29] Green B., Nachtigal D. (2013). The effects of temperature on sequential and mixture interactions between sucrose and saccharin. Chem. Senses.

[30] Kim M.J., Lee S.B., Lee H.S., Lee S.Y., Baek J.S., Kim D., Moon T.W., Robyt J.F., Park K.H. (1999). Comparative study of the inhibition of alpha-glucosidase, alpha-amylase, and cyclomaltodextrin glucanosyltransferase by acarbose, isoacarbose, and acarviosine-glucose. Arch. Biochem. Biophys..

[31] Yoon S.-H., Robyt J.F. (2003). Study of the inhibition of four alpha amylases by acarbose and its 4IV-α-maltohexaosyl and 4IV-α-maltododecaosyl analogues. Carbohydr. Res..

[32] Oppenheim F.G., Salih E., Siqueira W.L., Zhang W., Helmerhorst E.J. (2007). Salivary Proteome and Its Genetic Polymorphisms. Ann. N. Y. Acad. Sci..

[33] Hoebler C., Karinthi A., Devaux M.F., Guillon F., Gallant D.J.G., Bouchet B., Melegari C., Barry J.L. (1998). Physical and chemical transformations of cereal food during oral digestion in human subjects. Br. J. Nutr..

[34] Mandel A.L., Peyrot des Gachons C., Plank K.L., Alarcon S., Breslin P.A.S. (2010). Individual Differences in AMY1 Gene Copy Number, Salivary α-Amylase Levels, and the Perception of Oral Starch. PLoS ONE.

[35] Green B.G., Shaffer G.S., Gilmore M.M. (1993). Derivation and evaluation of a semantic scale of oral sensation magnitude with apparent ratio properties. Chem. Senses.

[36] Hartley C., Keast R.S.J., Bredie W.L.P. (2024). Investigating the Effect of Maltodextrins and Degree of Polymerisation on Individual Complex Carbohydrate Taste Sensitivity. Food Sci. Nutr..

[37] Purves D., Augustine G.J., Fitzpatrick D. (2001). Taste Perception in Humans.

[38] Henney J.E., Taylor C.L., Boon C.S. (2010). Taste and Flavor Roles of Sodium in Foods: A Unique Challenge to Reducing Sodium Intake.

[39] Deck C.M., Behrens M., Wendelin M., Ley J.P., Krammer G.E., Lieder B. (2022). Impact of lactisole on the time-intensity profile of selected sweeteners in dependence of the binding site. Food Chem. X.

